# Occupational Health Risk Assessment for Wastewater Treatment and Reuse in Kanpur, India

**DOI:** 10.3390/ijerph20126072

**Published:** 2023-06-07

**Authors:** Folake Monsurat Babalola, Lena Breitenmoser, Claire Furlong, Paul Campling, Christine Maria Hooijmans

**Affiliations:** 1 IHE Delft, Institute for Water Education, Westvest 7, 2611 AX Delft, The Netherlands; f.babalola@un-ihe.org (F.M.B.);; 2 Institute for Ecopreneurship, School of Life Sciences, University of Applied Sciences and Arts Northwestern Switzerland (FHNW), Hofackerstrasse 30, 4132 Muttenz, Switzerland; 3 VITO, Vlaamse Instelling voor Technologisch Onderzoek, Boeretang 200, 2400 Mol, Belgium

**Keywords:** agricultural reuse, constructed wetland plus, integrated permeate channel membrane, sanitation safety planning, semi-quantitative risk assessment, technological control measures

## Abstract

The treatment and reuse of wastewater for irrigation can lead to occupational health risks for sewage treatment plant (STP) workers and farmers. Sanitation Safety Planning (SSP) is an approach which can be used to measure and mitigate these risks. This paper explores what impact a novel secondary treatment process, consisting of an integrated permeate channel (IPC) membrane combined with a constructed wetland plus, has on the occupational health risks compared with the existing activated sludge wastewater treatment process and reuse system in Kanpur, Uttar Pradesh. A mixed methodology was used, which included key informant interviews, structured observations, and *E. coli* analysis. This data was used to undertake semi-quantitative risk assessments following the SSP approach. The novel secondary treatment increased the number of health risks which the STP workers were exposed to, but the severity of the risks was lower. This was due to the differences in treatment processes and infrastructures. The number of health risks for the farmers decreased both in number and severity. For their children, the severity of the health impacts decreased. These changes were due to the increase in the microbiological quality of the irrigation water. This study highlights the potential of using a semi-quantitative risk assessment to assess the occupational health impacts of using novel treatment technologies.

## 1. Introduction

Indian municipalities struggle to manage high water demands from households, industries, and agriculture [1]. Due to extensive aquifer and surface water use to meet these demands, particularly for irrigation, the groundwater levels have depleted rapidly, which is now one of the major water resource challenge India is facing [2,3]. To meet future demand-supply gaps, India needs to use water in a more efficient way and also focus on water reuse from treated wastewater to complement the capacities of the existing water supply infrastructures [4].

Recent estimates indicate that 63% of Indian municipal wastewater (also called ‘sewage’) is discharged without treatment [5]. A large part of the treated sewage, i.e., from 35 to 50% of all sewage treatment plants (STPs), does not meet the effluent discharge standards due to poorly performing sewage treatment plants [6]. This can lead to environmental and public health risks, especially if untreated or partially treated sewage is reused for irrigation. Several studies have reported community and occupational health risks for farmers and STP workers, who are exposed to untreated or partially treated wastewater [7,8,9,10]. Health risks relate to pathogens (disease-causing organisms) such as bacteria, viruses, protozoa, and helminth eggs. These pathogens cause diarrhoea, cholera, hepatitis, dysentery, and helminth egg infections [11]. The handling and reuse of untreated or partially treated wastewater in agriculture put STP workers and farmers at high risk of microbial infections due to the pathogen load [8,9]. Toxic chemicals and other hazardous substances in wastewater (including heavy metals such as chromium and cadmium, toxins, and sharp objects) also pose a risk to STP workers and farmers [12]. Additionally, accidents, ergonomic conditions, and psychological stress are frequent health risks for these exposure groups [13].

To address and mitigate health risks related to wastewater handling and reuse, the WHO 2006 Guidelines on the Safe Use of Wastewater, Excreta, and Greywater and the Sanitation Safety Planning (SSP) approach [11,12] were developed. These documents guide local authorities, wastewater utility managers, sanitation enterprises, farmers, and community-based organisations through a step-by-step management approach to protect public health within defined system boundaries [12]. The management approach entails the systematic identification of health risks in the defined system and guidance on control measures to be implemented to minimise adverse health impacts. The SSP process is co-created, i.e., is designed as a participatory action, allowing stakeholders from different sectors to prioritise health risks and agree on control measures and monitoring.

Several researchers have used the SSP risk-based management guidance to assess health risks related to on-site sanitation systems or centralised wastewater treatment systems in low- and middle-income settings. No such study was found for India. Health risks were assessed for different exposure groups in six studies, such as (i) wastewater treatment plant workers and peri-urban farming communities reusing untreated or partially treated effluents in Hanoi, Vietnam [8] and Iringa, Tanzania [10], (ii) faecal sludge emptiers and sludge users in Iringa, Tanzania [14] and Cap-Haitien, Haiti [15], and (iii) exposure groups in the sanitation and wastewater system in Salta, Argentina [16] and Kampala, Uganda [17]. Of these studies, two used the SSP semi-quantitative health risk matrix for the identification of the highest health risks stemming from microbial, chemical, and physical hazards and related hazardous events [14,18]. The study from Haiti [18] used data from regular *E.coli* monitoring of faecal-sludge-derived compost for microbial risk assessments. Frattarola et al. used a mixed health risk assessment approach, combining SSP’s semi-quantitative matrix with operational and maintenance procedure scoring to identify hazardous events in plant operation [10]. Clavijo et al. combined SSP with water safety planning and adapted the semi-quantitative risk levels to a percentage scale [16]. Frattarola et al. and Clavijo et al. used a simplified matrix, classifying risks according to hazardous events without further distinction of the hazard (i.e., biological, chemical, or physical) or the exposure pathways, and with fewer levels for likelihood and severity scores [10,16]. Fuhrimann et al. monitored pathogen indicators (*E. coli*, *Salmonella*, and Helminth eggs) along the wastewater and sanitation treatment and reuse chains as input variables for quantitative microbial risk assessment modelling [8,17]. Four of the studies used the health risks assessment to identify the highest health risks in the systems and recommended low-cost behavioural and technological control measures, e.g., for improving operation and maintenance procedures [8,10,14,17,18]. In the study in Salta, Argentina, it is not clear which specific control measures were identified, but the theoretical impact of all control measures addressing a specific hazardous event was calculated [16].

This study conducts a semi-quantitative health risk assessment, following the WHO’s SSP approach, for Indian STP workers, farmers and their children living in the wastewater-irrigated area of Kanpur, Uttar Pradesh. The study considers technological control measures. It evaluates the theoretical impact of a novel wastewater treatment technology on the number and level of health risks occurring in the current wastewater treatment and reuse system. This is different to the other outlined studies, which assessed the theoretical impacts of behavioural control measures or improved operation and maintenance procedures but not of alternative treatment trains. The aim of this study is thus to explore how a novel technology impacts the occupational and community health risks associated with the existing wastewater treatment technology and reuse system in Kanpur, India.

## 2. Materials and Methods

### 2.1. Study Background

This study was part of the Pavitra Ganga project, supported by the Indian government and the European Union under the Horizon 2020 research and innovation programme. It aims to support Sustainable Development Goal 6 (SDG6) by unlocking the environmental and economic potential of municipal wastewater treatment and reuse solutions for urban and peri-urban areas in India. The project demonstrates several novel treatment and reuse technologies, including a photo-activated sludge process, self-forming dynamic membrane bioreactors, and constructed wetland plus, a vertical-flow constructed wetland combined with adsorptive elements and specific sorbents [19].

### 2.2. Case Study Area

Kanpur Metropolitan Area (KMA) is among India’s most populated cities, with a population of approximately three million people [20]. It is situated in the centre of Uttar Pradesh on the south bank of the River Ganges, at 26.44° north and 80.33° east [21,22]. The region is among the Ganges’ most important urban and industrial areas [22]. KMA has a sub-tropical climate with three seasons experienced yearly: summer (with <30% humidity and high temperature of about 41 °C between March and May), monsoon (known as the wet season, with about 90% (700 mm) precipitation and temperature of about 27–35 °C between June and September); and winter (known as the cold season with temperature as low as 4–8 °C) [22].

In KMA, about 55% of urban households use off-site (sewered) sanitation systems, and only 41% of municipal wastewater generated (known locally as ‘sewage’) is conveyed to sewage treatment plants (STPs) [23]. There are six STPs in KMA, and this study focuses on Jajmau STP, one of the largest STPs in Kanpur, which treats 130 MLD of municipal wastewater [24]. After secondary treatment by an activated sludge process, the STP effluent is blended with industrial effluent from a common effluent treatment plant (CETP) and delivered to approximately 2500 ha of peri-urban agricultural land through a 4 km concrete irrigation channel [20,25]. Forty villages have access to these irrigation channels [26]. The study focused on 2 villages, Alaulapur (180 households) and Kulgaon (450 households), which use effluent from the STP and the CETP for irrigation from concrete (Alaulapur) and earthen channels (Kulgaon) to grow rice and wheat.

### 2.3. Pavitra Ganga Technologies

The novel technology explored in this paper is a combination of an integrated permeate channel (IPC) membrane in sequence with a constructed wetland plus (CW+). The IPC membrane aims to produce permeate with no suspended solids or coliforms, and very little organic matter [19,27], and CW+ aims to reduce excess nutrients and heavy metals [28,29]. The lab-scale IPC Membrane Filtration and CW+ were installed at the Indian Institute of Technology, IIT Kanpur. It should be noted that the lab-scale installations at IIT Kanpur were fed with primary effluent from Jajmau STP. At the end of the study period, pilot scale systems were installed at Jajmau STP [19].

### 2.4. E. coli Measurements

The study used *E. coli* as an indicator of the potential presence of faecal contaminants to assess the wastewater and irrigation water quality and the treatment efficiency of the novel technologies [30]. *E. coli* is proposed as indicator in the SSP Manual.

**Figure 1:**
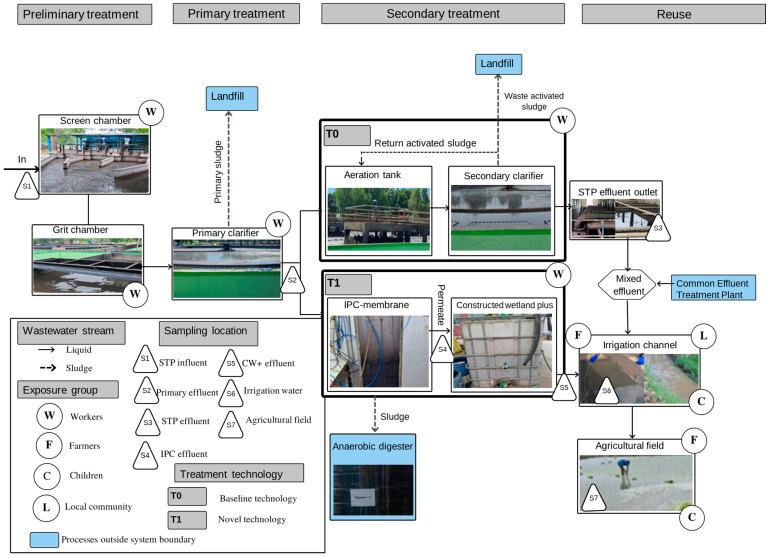
Process flow of wastewater treatment and reuse system in Kanpur, India. Figure shows baseline T0 and novel wastewater treatment technologies T1, the exposure groups identified, and the sampling locations. STP = sewage treatment plant; IPC = integrated permeate channel; CW+: = constructed wetland plus.

Sixty-eight grab samples of wastewater and treated effluents were obtained in duplicate (total n = 136) during four visits to five different sampling locations in Kanpur’s treatment and reuse system (Figure 1). All samples were taken between July and August 2022 in the monsoon season. Two additional sampling points were the lab-scale set-ups of the novel IPC-membrane and CW+ technologies. Each sample was collected using a 50 mL sterile plastic bottle and transported to the laboratory in a cooled, airtight box containing ice packs to keep the temperature at 4 °C [31]. The samples were examined and analysed for *E. coli* within 6 h after collection, following the standards described in Hardy Diagnostics [32].

Sample preparation and analysis were conducted at the IIT Kanpur laboratory. Serial dilutions of the samples were made aseptically [32]. The dilutions of 10^−2^ and 10^−3^ were plated on the Compact Dry^™^ EC plates [32] for samples obtained from the STP and reuse sites. The samples obtained from each of the novel technologies were plated without dilution. Samples were incubated for 24 h at a temperature of 35 ± 5 °C. The numbers of *E. coli* colonies were counted and recorded within 20–200 CFU [32]. Values < 20 CFU were recorded as too few to count (TFTC), and values > 200 CFU were recorded as too numerous to count (TNTC). The number of CFU per 100 mL was used for the statistical analysis, even if the number was below 20, as in the case of the effluent from the IPC-membrane and CW+. Analysis of variance (ANOVA) was used to explore the variation in *E. coli* concentrations related to the locations and time of visit. *p*-values ≤ 0.05 were considered to show a significant difference.

### 2.5. System Mapping and Health Risk Assessments

The study followed the system mapping and the semi-quantitative health risk assessment approach described in Modules 2 and 3 of the SSP Manual [12]. Data detailing the process flow for the system mapping were obtained from literature and Pavitra Ganga project reports. The secondary data were then validated with data obtained through key informant interviews (KIIs) and observations. All data collection was done between July and August 2022 during the monsoon season.

The participants were purposively selected based on their roles related to the STP and reuse of effluent for irrigation [12]. KIIs were undertaken with 11 farmers, three registered medical practitioners, two treatment plant workers, and two technology developers (IPC-membrane and CW+) using an interview guide [33]. All questions were discussed among the project partners and field assistants, who translated the questions written in English and responses from Hindi during the interview sessions when needed. Additionally, each interview was recorded, and notes were taken. All KII interviews were face-to-face and lasted between 15–60 min. Four visits to each study site were undertaken (to the STP, novel technologies, and effluent reuse sites), during which observations were undertaken, KIIs conducted and water samples collected. The observations were recorded using note-taking, photographs and an observational checklist adapted from the International Occupational Safety and Health Datasheets Standards [13].

A semi-quantitative risk assessment method was used to assess the health risks associated with wastewater treatment and reuse for irrigation [12]. Data obtained through the mapping process defined the exposure scenarios relating to wastewater treatment and effluent reuse. Data for hazard identification and existing control measures were obtained via KII and an observational checklist [13]. The exposure groups’ contact and interactions with the wastewater or effluent were observed to validate responses from the KIIs on the identified hazards, hazardous events, and existing control measures. *E. coli* data were used to determine the severity of the hazardous events related to microbial exposure. The risk was classified and scored using the method developed by the WHO (2015) to classify and score the severity and likelihood of identified hazards. Risks were categorised as low (L), medium (M), and high (H) based on these risk scores [12].

The risk assessments were conducted by compiling the data for identified hazards, control measures, and likelihood and severity of hazardous events in a risk matrix for different exposure scenarios. Exposure scenarios to workers include working at the existing STP (T0) and working with the novel technology (T1). Exposure scenarios to farmers and their children are effluent used from the existing STP (T0) for irrigation and effluent from T1 used for irrigation. The risk tables were revised and validated with relevant stakeholders from each context using a participatory approach. The complete risk tables can be found in the Supplementary Materials of this article. Then the outcomes of the health risk assessments of each exposure scenario to workers, farmers, and their children were compared for (i) the number of hazardous events and (ii) the health risk scores per hazardous event.

## 3. Results and Discussion

### 3.1. Mapping of the Wastewater Treatment and Reuse System

This study explores an off-site sanitation system (sewered sanitation), and the system boundaries are Jajmau STP and downstream reuse. The system includes treatment processes, liquid flows, and downstream villages where effluent is delivered through irrigation channels for reuse in agricultural fields (Figure 1). The exposure groups identified include (i) workers at STPs, (ii) farmers reusing the effluent for irrigation of their farmland and consuming crops produced with the contaminated effluent, (iii) local communities living close to irrigation channels which include the farmers and their families, and (iv) children playing along the irrigation channels. This study only considers the effluent due to its reuse and the STP workers, farmers, and children due to their high levels of exposure to raw wastewater or the effluent.

Figure 1 shows an overview of the STP treatment train and shows the three treatment stages (preliminary, primary, and secondary), the identified exposure groups, and the sampling locations. The preliminary and the primary treatment stages are the same for the baseline (T0) and novel technology (T1). The baseline secondary treatment is an activated sludge process (T0). The effluent of T0 is mixed with the effluent from a 36 MLD common effluent treatment plant (CETP) treating a mixture of tannery effluent and domestic wastewater at a ratio of 1:3 [5] and channelled through concrete irrigation canals for reuse on peri-urban agricultural land. All of the farmers interviewed in both villages (n = 11) reported using effluent from the irrigation channel to grow rice during the monsoon and wheat during the dry season. This is supported by another study that found 100% of farmers in Alaulapur (n = 18) and 85% in Kulgaon (n = 13) grow wheat and paddy with the STP effluent [26]. It was found that only 2 farmers (n = 11), 1 in each village, also used irrigation water to cultivate vegetables. In addition, 100% (n = 11) of the farmers interviewed reported that paths are dug to channel the irrigation water to their agricultural fields for flood irrigation. This method is described by Tilley et al. as suitable for farmers adopting labour-intensive irrigation practices to cultivate crops cooked before being eaten (e.g., wheat and paddy) [33]. It should be noted that this method was also used for the cultivation of vegetables, but it was not clear if they were cooked before consumption.

### 3.2. Microbiological Quality of Wastewater

**Figure 2:**
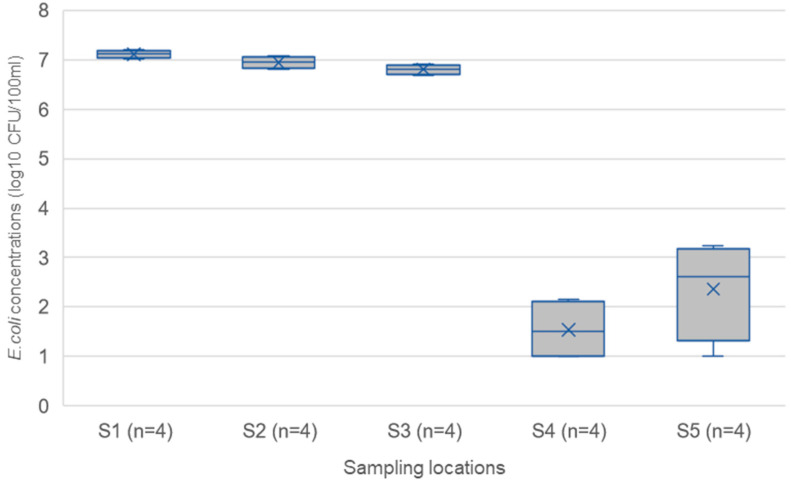
*E. coli* concentrations in influents and effluents (S1, S2, S3, S4, S5) of the baseline and novel treatment technologies as described in Figure 1. The lower and upper box boundaries are the 25th and 75th percentiles, respectively, the line inside the box is the median, the cross inside the box is the mean, and the lower and upper error lines are the 5th and 95th percentiles. S1 = influent preliminary treatment, S2 = effluent primary treatment, S3 = effluent activated sludge secondary treatment T0, S4 = effluent IPC membrane secondary treatment T1, and S5 = effluent CW+ secondary treatment T1.

Figure 2 shows the mean *E. coli* concentrations of influents (S1 = 7.1 log_10_ CFU/100 mL), primary effluents (S2 = 6.9 log_10_ CFU/100mL), and secondary effluents of T0 and T1 (sampling locations S3, S4, and S5 in Figure 1). The treatment efficiency of T0 for *E. coli* is only 51% with <1 log_10_ reduction, which was not found to be a significant reduction (ANOVA = 0.8449: *p* > 0.05). A greater reduction in *E. coli* was expected, as between 2.0 and 2.5 log reduction can be found in a well-functioning activated sludge process [34]. The mean *E. coli* concentrations (6.51 × 10^6^ CFU/100 mL) of the secondary effluents from T0 show non-compliance with the Indian effluent discharge standards, which is 230 MPN/100 mL for faecal coliforms, equal to 230 CFU/100 mL [35]. The average *E. coli* concentrations obtained from the secondary effluents of both T1 technologies (the mean *E. coli* concentrations of IPC Membrane Filtration = 11 CFU/100 mL and CW+ = 130 CFU/100 mL) meet the Indian effluent discharge standards of 230 MPN/100 mL [35]. An increased level of *E. coli* concentration in S5, the CW+ effluent, could be attributed to cross-contamination from birds or other warm-blooded animals capable of excreting into the exposed effluent in the open environment [36]. The overall treatment efficiency of T1 (>6 log reduction) was high compared to the baseline technology T0 (<1 log reduction). Furthermore, the log reduction (>6 log reduction) was above the required limit (4 log_10_) set for labour-intensive irrigation [11].

**Figure 3:**
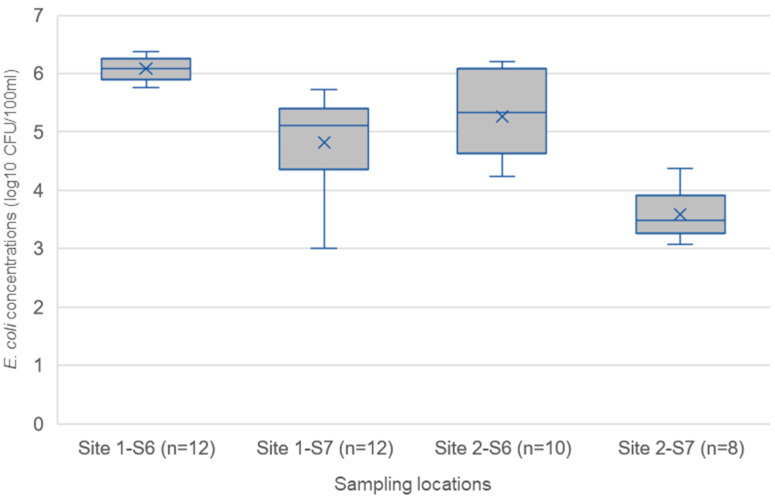
*E. coli* concentrations in irrigation water from irrigation channels (S6) and agricultural fields (S7) at sites 1: Alaulapur and 2: Kulgaon. The lower and upper box boundaries are the 25th and 75th percentiles, respectively, the line inside the box is the median, the cross inside the box is the mean, and the lower and upper error lines are the 5th and 95th percentiles.

Figure 3 shows *E. coli* concentrations of irrigation water from the irrigation channels (S6) and the agricultural fields (S7) of the two sites reusing the treated effluents for irrigation. The irrigation water from S6 and S7 show *E. coli* mean concentrations of 6.1 and 4.8 log_10_ CFU/100 mL, respectively. This range is similar to the findings of a study in Hanoi, Vietnam, which found mean *E. coli* concentrations of 6.0 log_10_ CFU/100 mL of wastewater reused in agricultural fields, but they attributed the high concentration of *E. coli* in their study to open defecation and animal waste [8]. There was a significant difference between the irrigation water quality from S6 and S7 in Alaulapur (S1) (ANOVA = 0.0108: *p* < 0.05), indicating a reduction in the concentration of *E. coli* in the field irrigation water compared to the irrigation channels. A similar pattern was found in Kulgaon (S2), where the irrigation water from S6 and S7 showed *E. coli* mean concentrations of 5.3 and 3.6 log_10_ CFU/100 mL, but this was not found to be a significant (ANOVA = 0.4414: *p* > 0.05) reduction, since the site data points significantly overlap. Kulgaon has lower *E. coli* concentrations in the irrigation channels and the fields compared to Alaulapur, but this difference was not found to be significant (ANOVA = 0.9918: *p* > 0.05). The *E. coli* concentrations detected in the water being used for irrigation in the field at both sites were above the limit of 4 log reduction for safe reuse in labour-intensive irrigation suggested by WHO 2006 [11], such as flood irrigation which is being used in Kulgaon and Alaulapur. It should be noted that this study was undertaken during the monsoon season, so there was a dilution of all irrigation waters by rainfall. This means that the irrigation waters will probably have even higher concentrations of *E. coli* during the post-monsoon seasons.

### 3.3. Health Risks Assessments

**Figure 4:**
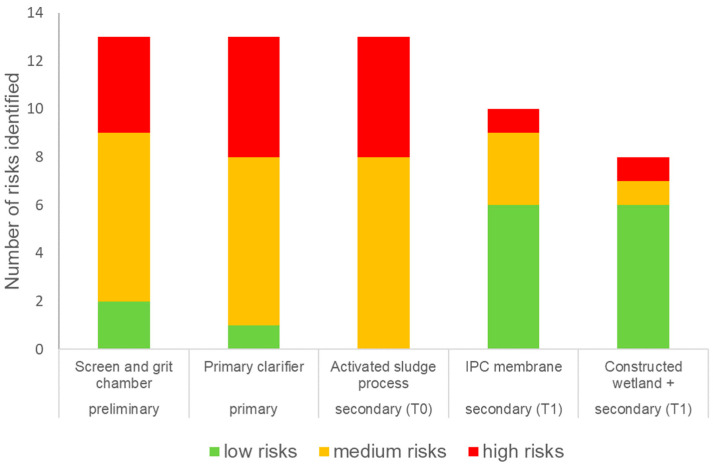
Comparing the health risk levels for STP workers associated with the STP and novel technology treatment processes.

The health risk assessment for the STP workers, farmers and their children was conducted for the four exposure scenarios, namely workers exposed during the treatment of wastewater with either T0 or T1 as a secondary treatment process, and farmers and children exposed to effluent from either T0 or T1. Twelve hazardous events and hazards for treatment plant workers were identified across the treatment processes T0 and T1 through interviews with the workers and using an observational checklist [13]. The hazards identified were categorised into four main groups A: exposure to harmful gases, B: accidents, C: diseases, and D: exposure to high noise levels (Table 1). The risk categories are displayed in Figure 4, and the scores for likelihood, severity, and risks are given in the Supplementary Materials to this article.

In Category A, STP workers were found to be exposed to harmful gases, such as hydrogen sulphide (H_2_S), produced during anaerobic degradation of organic matter in places such as pits, manholes and tunnels, and could be identified by its distinctive malodour [37]. H_2_S can be very hazardous to STP workers leading to sudden unconsciousness or death at high levels (>1000 ppm). Lower levels of H_2_S with repeated exposure lead to fatigue, loss of appetite, headache, poor memory, or dizziness [37]. Workers could be at risk of these hazards during routine operation and maintenance, such as monitoring and cleaning activities at the STP. Medium risks were identified at the preliminary, primary, and secondary (T0) treatment stages due to malodour and H_2_S. In comparison, low risks were identified for the secondary treatment (T1, IPC Membrane Filtration), which uses an enclosed filtration process with automated backwashing processes, thus reducing the exposure of STP workers to malodour or potential H_2_S formed during the degradation of the cake layer. CW+ receives influent with low organic matter and is an open system; thus, no risks for H_2_S were found. Workers could also be exposed to aerosols and potential aerosol-based pathogen transmission through inhalation. Low risks were identified from the preliminary and primary stages, whereas the secondary treatment (T0) posed medium risks for aerosol-pathogen transmissions due to the aeration-based treatment process [38]. Aerosols were not identified as a hazard in T1, as treatment occurs in an enclosed membrane reactor, and the CW+ uses a trickle method to distribute the influent over the wetland [29] so aerosols are not produced and the influent is pathogen-free (Figure 2).

Five health hazards were identified in Category B (accidents). The risks associated with falls, slips, and cuts are high during the preliminary, primary, and secondary treatment (T0) processes. STP workers are likely to slip due to spillages from open tanks and by coming into contact with sharp objects during daily inspection or sample collection. Low risks were identified for the IPC Membrane Filtration treatment process (T1) as workers have no interaction with mechanical infrastructures or sharp objects in the membrane reactor. High risks were identified for CW+ systems (T1) which requires sharp objects for the management of biomass growth. The risks of electric shocks were high for workers during the primary, activated sludge (T0) treatment steps and for the IPC Membrane Filtration treatment processes (part of T1) due to the potential exposure of workers to electricity in a wet environment. For the CW+ process (part of T1), which does not use electricity, there was no risk. Suffocation associated with secondary treatment process T0 posed medium risks to the workers when carrying out daily inspection or installation and maintenance works in confined places such as tanks, tunnels, or pits. In contrast, there are no risks of suffocation during secondary treatment processes (T1), where operations are carried out in the open. The risks associated with drowning were high during primary and secondary treatment (T0) due to exposure to an open clarifier and aeration tanks even though there were handrails. Although the likelihood of falling into these open tanks was low, the severity of the outcome would be high, e.g., death. No risk of drowning was identified for preliminary or T1 as the operators are not exposed to liquids in an open system.

In Category C, medium risks were identified for diseases stemming from exposure to microbial pathogens or skin irritants and musculoskeletal disorders at the preliminary, primary, and secondary T0 stages. At the same time, mosquito bites pose a high risk to workers during these processes due to open vessels with stagnant water that are potential mosquito-breeding sites. Medium risks were identified for the IPC Membrane Filtration through exposure to microbial pathogens or skin irritants, as workers could accidentally ingest pathogens or absorb skin irritants while cleaning the membrane. The influent for the CW+ is pathogen-free (Figure 2), so although this system was open, no pathogen risk was associated with contact with this influent. The only medium risk was identified for mosquito bites, as the CW+ uses a trickling system to distribute water across the wetland, so stagnant surface water pools could form.

In Category D (exposure to high noise level), workers were found to be exposed to medium risks of noise for the preliminary, primary, and secondary treatment (T0) and IPC-membrane, generated from electromechanical equipment such as motors and air compressors [27,39]. This was not the case for CW+ which does not require such equipment for its operation.

Figure 4 shows that 13 health risks were identified for the current treatment train processes, the preliminary (2 low, 7 medium, 4 high), primary (1 low, 7 medium, 5 high), and for secondary treatment T0 (0 low, 8 medium, 5 high) processes, whereas 10 risks were identified for the IPC Membrane Filtration (6 low, 3 medium, 1 high) and 8 for the CW+ (6 low, 1 medium, 1 high). In total, T1 had 18 risks (12 low, 4 medium and 1 high), a higher number of risks compared to the secondary treatment of T0 (13 compared to 18), but the severity of the risks was lower. There were fewer medium and high health risks in T1 (4 medium and 2 high) compared to T0 (8 medium and 5 high). This was due to the risk associated with the different treatment processes and their associated infrastructure. The study was undertaken during the monsoon season, but during the post-monsoon season when there is less rainfall, there may be a higher pathogen load in the wastewater, which could increase some of the risks associated with microbial pathogens.

This study also assessed health risks for farmers and children downstream of the STP (T0 and T1 effluent reuse). No distinction was made between the two villages for the health risk assessments as irrigation practices and *E. coli* concentrations in the irrigation water (Figure 3) used were similar. Hence only one risk assessment was conducted for both sites. In Table 2, 11 hazardous events and hazards were identified for farmers and 8 for children exposed to treated effluents on farmlands through observations [12], and interviews with farmers (n = 11) and registered medical practitioners (n = 3) for both villages. The hazards identified were categorised into only three main groups (A: exposure to harmful gases, B: accidents, and C: diseases) as farmers and their children were not exposed to high noise levels (Category D for the STP workers).

In Category A, a medium risk of exposure to malodour from the effluent reuse (T0) was identified for the farmers and children in both villages, whereas low risks were envisaged if effluent from T1 was used for irrigation.

The health hazards and hazardous events identified in Category B were falls and slips in both exposure scenarios (reusing T0 and T1). A high risk of exposure was identified for farmers and children to fall or slip in the wet fields during farming activities due to their irrigation method. This could result in severe injuries such as bone fractures or pulled muscles. In Category C, the diseases identified in the study were attributed to five health hazards, including C1: microbial pathogens, C2: soil helminths, C3: skin irritants, C4: vector-related diseases, and C5: musculoskeletal disorder [12]. The farmers were exposed to irrigation water during farming activities, including when digging paths to channel irrigation water to the field. Children were exposed to irrigation water while playing and helping their parents in the fields. The farmers and families all reported consuming crops (wheat and paddy) grown with treated effluent (100%, n = 11). This is supported by a previous study that found that farmers from both sites consume wheat and paddy grown with irrigation water [26]. The farmers were therefore exposed to potential pathogens when growing, harvesting, and preparing wheat and rice. These exposures can lead to faecal–oral diseases such as diarrhoea and soil helminth infections [40]. The exposure routes identified for hazardous events and health hazards C1–C4 are ingestion and skin absorption, with symptoms such as cold, fever, joint pain, nausea, vomiting, stomach pain, diarrhoea, and skin irritation (red spots on hands and legs) reported by registered medical practitioners. Other studies have also reported the prevalence of similar symptoms among farmers reusing treated effluent for irrigation in this area [41] and in other locations in India [41,42]. Additional health hazards, such as hookworms (Category C2), seem likely for farmers and children in the area [43,44] but were not specifically raised during KIIs.

A high risk of exposure to health hazards in Category C1 and C2 was identified during the farming activities, as none of the farmers was observed wearing personal protective equipment such as boots, facemasks, and gloves. The farmers, therefore, had direct contact with the irrigation water, which could lead to faecal–oral diseases due to the poor microbiological quality (Figure 3). A medium risk in Category C3 was identified, leading to itching and rashes, as reported by most farmers, due to skin contact with the irrigation water. The microbiological quality of the irrigation water will probably decrease during the post-monsoon season, when rainfall is reduced, since rainwater was probably diluting the effluent from the STP during the study. This means that the risk for the farmers and their children related to microbial pathogen exposure will probably increase post-monsoon. In Category C4 (vector-related diseases), a high risk was identified for farmers and children through mosquito bites, as dengue fever and malaria are endemic in Kanpur [45,46] and due to their use of flood irrigation which can create places for mosquito breeding. A high risk of exposure to musculoskeletal disorder (Category C5) was identified for the farmers due to frequent bending and kneeling during farming activities [41], whereas a low risk was identified for children as the activities of the children on the field may not significantly cause such a disease.

**Figure 5:**
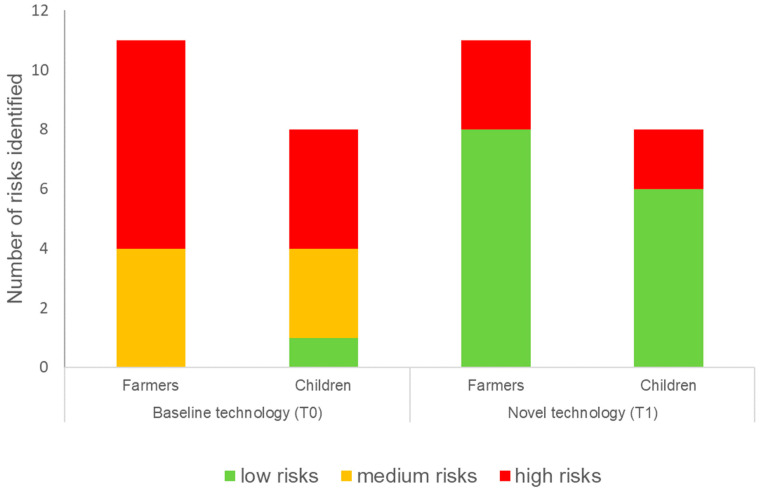
Comparing the health risk levels of farmers and children without (baseline technology T0) and with technological control measures (novel technology T1).

The impacts of implementing T1 as an alternative to T0 can be seen in Table 2 and Figure 5. For T0, the farmers were exposed to 11 risks (0 low, 4 medium, 7 high). Farmers are exposed to the same number of risks for T1, but the magnitude of 8 risks are reduced (8 low, 0 medium, 3 high). Children are exposed to a lower number of risks compared to farmers for T0 8 risks compared to 11 in T1. This is because children are less frequently in the fields compared to the farmers, e.g., only 45% (n = 11) of farmers in both villages reported that their children accompanied them to the fields and the tasks the farmers undertake. For T0, the children were exposed to 8 risks (1 low, 3 medium and 4 high), and, as with the farmers for T1, the number of risks remains the same, but the magnitude is reduced (6 low, 0 medium, 2 high).

The reduction in risk is associated with the increased effluent quality from T1, as this will lead to no or negligible health effects (Figure 2). If the activated sludge system (T0) was functioning better, there would be a reduction in microbial load in the irrigation water by 2 logs, but as the overall concentrations of *E. coli* would still remain high (approximately 5 log_10_ CFU/100 mL), and above the recommendations for labour intensive agriculture [11], the risks associated with this hazard would still be high. Risks associated with vector-related diseases and musculoskeletal disorders (Category C4 and C5) remain high for the farmers, as they are not directly linked to the effluent water quality. This is because the practices are not changed.

## 4. Study Limitations

It should be noted that this study has several limitations. The system mapping and microbiological water quality monitoring does not consider point source pollution in the irrigation channels, such as open defecation or animal faeces, which other studies have found to significantly contribute to *E. coli* levels in irrigation waters [8]. *E. coli* was used as an indicator for faecal contamination despite its acknowledged limitations. This study only considers the effluent from the STP as sludge is taken to landfill, and the risk associated with this activity and possible reuse could be explored in a further study. Kanpur is a highly industrial city, and the irrigation water is known to contain industrial effluent. This study did not consider any risk associated with chemical pollutants such as chromium, which is known to be an issue in this area [25,47]. Nor did the study consider *E. coli* levels in the CETP effluent, although it should be noted that the volume of this effluent stream was approximately one-third of that coming from the STP. The study was undertaken during the monsoon season, which means that the effluent from the STP and the irrigation water will be diluted by precipitation, so it is thought that risks associated with contact with the effluent or irrigation water will increase post-monsoon. The novel technologies (T1) are currently in a laboratory set-up, and the developers may not be fully aware of all risks associated with the full-scale operation. Additionally, the technology infrastructure may differ from what was envisaged by the developers. The system mapping and health risk assessments were conducted based on a small number of KIIs and observations rather than using a participatory process with iterative consultation rounds and including all relevant stakeholders in the system. It is likely that not all hazards and hazardous events have been identified due to stakeholder selection bias and the risk that rating was subjected to confirmation (interviewer) and reporting bias (interviewees).

## 5. Conclusions

The levels of *E. coli* found in the effluent at the sewage treatment plant in Kanpur were surprisingly high, as the system was achieving under 1 log removal. The theoretical impact of swapping of a novel secondary treatment (IPC Membrane Filtration and CW+) for the current activated sludge process increased the number of health risks to which the sewage treatment plant operators were exposed, but the severity of the risks was lower. This was due to the different treatment processes and their associated infrastructure. The estimated health impacts on the farmers who were exposed to the STP effluent used as irrigation water decreased as the number and severity of the risks decreased, whereas for their children, the number of health impacts remained the same, but the severity decreased. This change was due to the increase in the microbiological quality of the irrigation water (effluent). This impact would be the same if the activated sludge process was performing up to the expected 2 log removals. There would still be high levels of *E. coli* in the irrigation water and thus high related microbial health risks during irrigation and farming activities. This study shows the potential of using a semi-quantitative occupational health risk assessment to assess the impacts of using a new technology or alternative sewage treatment technologies. The authors believe this is the first time this has been carried out. Additionally, this is the first time a semi-quantitative occupational health risk assessment was conducted for an STP in India. Using this risk-based management approach could be beneficial to STP operators and employers who need to provide and maintain a working environment that safeguards the health of their employees as required by the new Indian occupational safety, health and working conditions Code launched in 2020.

## Figures and Tables

**Table 1:** Health risk assessments for workers at sewage treatment plants.

Main Hazard Categories Identified	Risk Levels Identified				
	Preliminary	Primary	Secondary (T0)	Secondary (T1)	
	Screen and Grit Chamber	Primary Clarifier	Activated Sludge Process/Secondary Clarifier	IPC Membrane	Constructed Wetland +
A. Exposure to harmful gases					
A1. Malodour	medium	medium	medium	low	low
A2. Hydrogen sulfide	medium	medium	medium	low	low
A3. Aerosols	low	low	medium	none	none
B. Accidents					
B1. Falls, slips	high	high	high	low	low
B2. Cuts	high	high	high	low	high
B3. Electric shock	high	high	high	high	none
B4. Suffocation	medium	medium	medium	none	none
B5. Drowning	low	high	high	none	none
C. Diseases					
C1. Through microbial pathogens	medium	medium	medium	medium	low
C2. Through mosquito bites	high	high	high	low	medium
C3. Skin irritation	medium	medium	medium	medium	low
C4. Musculoskeletal disorders	medium	medium	medium	low	low
D. Exposure to high noise level					
D1. Noise	medium	medium	medium	medium	none

Colour codes: grey = no risk; green= low risk, orange = medium risk, red = high risk.

**Table 2:** Health risk assessments of farmers reusing irrigation water and children playing alongside irrigation channels.

Main Hazard Categories Identified	Risk Levels Identified			
	Baseline Technology (T0)		Novel Technology (T1)	
	Farmers	Children	Farmers	Children
A. Exposure to harmful gases				
A1. Malodour	medium	medium	low	low
B. Accidents				
B1. Falls, slips	high	high	high	high
C. Diseases				
C1. Microbial pathogens				
During flood irrigation	high	none	low	none
During farming activities	high	none	low	none
Through playing and helping parents	none	high	none	low
During preparation of contaminated crop	medium	medium	low	low
C2. Soil helminths				
During irrigation	high	none	low	none
During farming activities	high	none	low	none
Through playing and helping parents	none	high	none	low
C3. Skin irritation				
During irrigation	medium	none	low	none
During farming activities	medium	none	low	none
Through playing and helping parents	none	medium	none	low
C4. Vector-related diseases				
Through mosquito bites	high	high	high	high
C5. Musculoskeletal disorders				
Through farming activities	high	low	high	low

Color codes: grey = no risk; green= low risk, orange = medium risk, red = high risk.

## Data Availability

The data presented in this study is available within the article and Supplementary Material to this article.

## Supplementary Materials

The following supporting information can be downloaded at: https://www.mdpi.com/article/10.3390/ijerph20126072/s1. Table S1: Description for classifying likelihood and severity in risk assessments [12], Table S2: Matrix for semi-quantitative risk assessment [12], Table S3: STP and novel treatment processes risk assessment table, and Table S4: Reuse scenario risk assessment table.
