# Supplementary material for: Occupational Health Risk Assessment for Wastewater Treatment and Reuse in Kanpur, India
Source: Int J Environ Res Public Health. 2023 Jun 7;20(12):6072. doi: 10.3390/ijerph20126072 (PMC10298189; doi:10.3390/ijerph20126072)
Supplement: Supplementary file 1 [file ijerph-20-06072-s001.zip › ijerph-2399155-supplementary.pdf]

Table S1: Description for classifying likelihood and severity in risk assessment (source: WHO, 2015).

Descriptor		Description
**Likelihood (L)**		
**1**	Very unlikely	It has not happened in the past, and it is **highly improbable** it will happen in the next 12 months (or another reasonable period).
**2**	Unlikely	It has not happened in the past but **may occur in exceptional circumstances** in the next 12 months (or another reasonable period).
**3**	Possible	It May have happened in the past and **may occur under regular circumstances** in the next 12 months (or another reasonable period)
**4**	Likely	It has happened in the past and **is likely to occur** in the next 12 months (or another reasonable period)
**5**	Almost certain	It has happened in the past and **will almost certainly occur** in the next 12 months (or another reasonable period)
**Severity (S)**		
**1**	Insignificant	Hazard or hazardous event resulting in **no or negligible health effects** compared to background levels.
**2**	Minor	Hazard or hazardous event potentially resulting in **minor health effects** (e.g. temporary symptoms like irritation, nausea, headache)
**4**	Moderate	Hazard or hazardous event potentially resulting in **self-limiting health effects or minor illness** (e.g. acute diarrhoea, vomiting, upper respiratory tract infection, minor trauma).
**8**	Major	Hazard or hazardous event potentially resulting in **illness or injury** (e.g. malaria, schistosomiasis, food-borne trematodiases, chronic diarrhoea, chronic respiratory problems, neurological disorders, bone fracture); and may lead to a legal complaint; and or significant regulatory non-compliance
**16**	Catastrophic	Hazard or hazardous event potentially resulting in **serious illness or injury, or even loss of life** (e.g. severe poisoning, loss of extremities, severe burns, drowning); and will likely lead to a significant investigation by a regulator with a prosecution.

Table S2: Matrix for semi-quantitative risk assessment (WH0, 2015).

			SEVERITY (S)				
			**Insignificant**	**Minor**	**Moderate**	**Major**	**Catastrophic**
			1	2	4	8	16
**LIKELIHOOD (L)**	Very unlikely	1	1	2	4	8	16
	Unlikey	2	2	4	8	16	32
	Possible	3	3	6	12	24	48
	Likely	4	4	8	16	32	64
	Almost certain	5	5	10	20	40	80
**Risk Score R = (L) * (S)**			<6	7 - 12		13 - 32	>32
**RISK level**			Low Risk	Medium Risk		High Risk	Very High Risk

Table S3: STP and novel treatment processes risk assessment table.

Treatment process	Hazard Identification					Existing control measure	Risk Assesments L=Likelihood, S=Severity, R=Risk level			
	Category	Hazardous event	Hazard	Exposure route	Exposure group	Description	L	S	Score	R
Preliminary treatment (Screen & Grit chamber)	A	Exposure to hazardous gases when working in confined places	Hydrogen sulfide & malodor	Inhalation	Exposure scenario (E1) Workers (W)	Use of PPE such as face masks	5	2	10	M
			Aerosols				2	2	4	L
	B	Accidents from contact with sharp objects, electrical divices (naked wire) and spillages during daily inspection and sample collection	Falls, slips, cuts	Skin contact		Walkway with railing system	3	8	24	H
			Electric shock			Use of PPE such as gloves, boots	2	16	32	H
	C	Eposure to untreated sewage during operation and maintenance of the STP	Microbial pathogens, skin irritants	Ingestion and skin contact		Use of PPE such as face masks, gloves, boots; Regular hand washing	3	4	12	M
		Mosquito breeding in surface water	Vector-related diseases	Mosquito bites		Quarterly pest control	4	4	16	H
		Musculoskeletal disorder from taking uncomfortable postures during inspection and installation	Musculoskeletal disorder	Uncomfortable posture		None	3	4	12	M
	D	Exposure to high noise level from electro-mechanical infrastructure	Noise			Regular health checkup	5	2	10	M
Primary treatment (primary clarifier)	A	Exposure to hazardous gases when working in confined places	Hydrogen sulfide & malodor	Inhalation		Use of PPE such as face masks	5	2	10	M
			Aerosols				2	2	4	L
	B	Accidents from contact with sharp objects, electrical divices (naked wire) and spillages during daily inspection and sample collection	Falls, slips, cuts	Skin contact, accidental ingestion		Walkway with railing system	3	8	24	H
			Electric shock			Use of PPE such as gloves, boots	2	16	32	H
		Falling into the open clarifier	Drowning			Walkway with railing system	2	16	32	H
	C	Eposure to untreated sewage during operation and maintenance of the STP	Microbial pathogens, skin irritants			Use of PPE such as face masks, gloves, boots; Regular hand washing	3	4	12	M
		Mosquito breeding in surface water	Vector-related diseases	Mosquito bites		Quarterly pest control	4	4	16	H
		Musculoskeletal disorder from taking uncomfortable postures during inspection and installation	Musculoskeletal disorder	Uncomfortable posture		None	3	4	12	M
	D	Exposure to high noise level from electro-mechanical infrastructure	Noise			Regular health checkup	5	2	10	M
Secondary treatment (T0) activated sludge process and secondary clarifier	A	Exposure to hazardous gases when working in confined places	Hydrogen sulfide & malodor	Inhalation		Use of PPE such as face masks	5	2	10	M
			Aerosols				3	4	12	M
	B	Accidents from contact with sharp objects, electrical divices (naked wire) and spillages during daily inspection and sample collection	Falls, slips, cuts	Skin contact, accidental ingestion		Walkway with railing system	3	8	24	H
			Electric shock			Use of PPE such as gloves, boots	2	16	32	H
		Falling into the open clarifier	Drowning			Walkway with railing system	2	16	32	H
	C	Eposure to untreated sewage during operation and maintenance of the STP	Microbial pathogens, skin irritants			Use of PPE such as face masks, gloves, boots; Regular hand washing	3	4	12	M
		Mosquito breeding in surface water	Vector-related diseases	Mosquito bites		Quarterly pest control	4	4	16	H
		Musculoskeletal disorder from taking uncomfortable postures during inspection and installation	Musculoskeletal disorder	uncomfortable posture		None	3	4	12	M
	D	Exposure to high noise level from electro-mechanical infrastructure	Noise			Regular health checkup	5	2	10	M

	A	Exposure to hazardous gases when working in confined places	Hydrogen sulfide & malodor	Inhalation		Use of PPE such as face masks	1	2	2	L
	B	Accidents from contact with sharp objects, electrical divices (naked wire) and spillages during daily inspection and	Falls, slips, cuts				1	4	4	L
			Electric shock	Skin contact, accidental ingestion			1	16	16	H
		Eposure to untreated sewage during operation and maintenance of the STP	Microbial pathogens, skin irritants			Use of PPE such as gloves, facemasks	3	4	12	M
		Mosquito breeding in surface water	Vector-related diseases	Mosquito bites		Use of mosquito repellant is advised; cover the skin with loose clothes	1	4	4	L
	C	Musculoskeletal disorder from taking uncomfortable postures during inspection and installation	Musculoskeletal disorder	Uncomfortable posture			3	4	12	M
Secondary treatment (T1) IPC membrane	D	Exposure to high noise level from electro-mechanical infrastructure	Noise		Exposure scenario (E2) Workers (W)		5	2	10	M
	A	Exposure to hazardous gases when working in confined places	Hydrogen sulfide & malodor	Inhalation		Use of PPE such as gloves, boots	1	2	2	L
	B	Accidents from contact with sharp objects, electrical divices (naked wire) and spillages during daily inspection and sample collection	Falls, slips, cuts	Skin contact, accidental ingestion			2	8	16	H
		Eposure to untreated sewage during operation and maintenance of the STP	Microbial pathogens, skin irritants			Use of PPE such as gloves, facemasks	1	4	4	L
		Mosquito breeding in surface water	Vector-related diseases	Mosquito bites		Use of mosquito repellant is advised; cover the skin with loose clothes	1	4	4	L
Secondary treatment (T1) Constructed wetland +	C	Musculoskeletal disorder from taking uncomfortable postures during inspection and installation	Musculoskeletal disorder	Uncomfortable posture			3	4	12	M

Table S4: Reuse scenario risk assessment table.

Reuse Senario		Hazard Identification				Existing control measure	STP Risk Assesments L=Likelihood, S=Severity, R=Risk level				Novel Technology Risk Assesments L=Likelihood, S=Severity, R=Risk level			
	Category	Hazardous event	Hazard	Exposure route	Exposure group (F= Farmers, C= Children)	Description	L	S	Score	R	L	S	Score	R
Effluent used for irrigation	A	Exposure to hazardous gases	Malodor	Inhalation	F and C	None	5	2	10	M	2	2	4	L
	B	Accidents from falls and slips on a wet and slippery surface while working on the field	Falls, slips	Skin contact	F and C	None	3	8	24	H	3	8	24	H
	C1	Eposure during flood irrigation	Microbial pathogens	Ingestion and skin contact	F	None	5	4	20	H	1	4	4	L
	C2		Soil helminths	Skin contact	F	None	4	4	16	H	4	1	4	L
	C3		Skin irritants	Skin contact	F	None	5	2	10	M	5	1	5	L
	C1	Eposure during Farming activities	Microbial pathogens	Ingestion and skin contact	F	None	5	4	20	H	5	1	5	L
	C2		Soil helminths	Skin contact	F	None	4	4	16	H	4	1	4	L
	C3		Skin irritants	Skin contact	F	None	5	2	10	M	5	1	5	L
	C1	Eposure through playing and helping parents on field	Microbial pathogens	Ingestion and skin contact	C	None	5	4	20	H	5	1	5	L
	C2		Soil helminths	Skin contact	C	None	4	4	16	H	4	1	4	L
	C3		Skin irritants	Skin contact	C	None	5	2	10	M	5	1	2	L
	C1	Exposure during preparation of contaminated crops	Microbial pathogens	Ingestion and skin contact	F and C	Cooked before consumption	3	4	12	M	3	1	3	L
	C4	Mosquito breeding in irrigation water	Vector-related diseases	Mosquito bites	F and C	Use of pesticides	4	4	16	H	4	4	16	H
	C5	Musculoskeletal disorder from taking uncomfortable postures during farming activities	Musculoskeletal disorder	Uncomfortable posture	F	None	3	8	24	H	3	8	24	H
					C	None	1	4	4	L	1	4	4	L
